# Post-COVID-19 Fatigue and SARS-CoV-2 Specific Humoral and T-Cell Responses in Male and Female Outpatients

**DOI:** 10.3389/fimmu.2022.902140

**Published:** 2022-05-26

**Authors:** Christa Meisinger, Yvonne Goßlau, Tobias D. Warm, Vincenza Leone, Alexander Hyhlik-Dürr, Jakob Linseisen, Inge Kirchberger

**Affiliations:** ^1^ Epidemiology, Faculty of Medicine, University of Augsburg, Augsburg, Germany; ^2^ Vascular Surgery, Faculty of Medicine, University of Augsburg, Augsburg, Germany; ^3^ Institute for Medical Information Processing, Biometry and Epidemiology (IBE), Ludwig-Maximilians Universität München (LMU) Munich, Munich, Germany

**Keywords:** COVID-19, fatigue, outpatients, T-cells, ELISpot, inflammation

## Abstract

**Background:**

Information on the clinical characteristics and pathophysiological mechanisms underlying post-COVID-19 fatigue are scarce. The main objective of this study was to evaluate sex-specific humoral and T-cell responses associated with post-COVID-19 fatigue in a sample of individuals treated as outpatients.

**Methods:**

At a median time of 279 (179;325) days after the acute infection, a total of 281 individuals (45.9% men) aged 18-87 years old were included in the analysis. The participants were examined at the University Hospital of Augsburg, Southern Germany. Fatigue was assessed using the Fatigue Assessment Scale (FAS). Levels of anti-SARS-CoV2-spike IgG antibodies were measured by an enzyme-linked immunosorbent assay (ELISA), and for exploration of the SARS-CoV2-specific T-cell response, ex vivo ELISpot/FLUOROspot assays were conducted using an interferon-γ (IFN-γ) and interleukin-2 (IL-2) SARS-CoV-iSpot kit.

**Results:**

Women more significantly suffered from post-COVID-19 fatigue in comparison to men (47.4% versus 25.6%, p=0.0002). Females but not males with fatigue showed a significantly lower number of T-cells producing IFN-γ, IL-2 or both IL-2 and IFNγ in comparison with females without fatigue. In both sexes, serum levels of anti-SARS-CoV2-spike IgG antibodies did not differ significantly between participants with or without fatigue.

**Conclusions:**

Development of fatigue after acute COVID-19 disease might be associated with SARS-CoV-2-specific T-cell responses in women, but not men after a mild infection course treated outpatient.

## Introduction

Chronic fatigue syndrome is characterized by unusual fatigue, a significant lack of energy, and a persistent feeling of exhaustion lasting for more than six months (1). Chronic fatigue is a phenomenon common to conditions such as cancer, neurological conditions such as stroke and multiple sclerosis, and diseases of the immune system, and it affects up to 18% of the population in Western countries (1). Patients with fatigue syndrome are predominantly female, and in prior studies, chronic subclinical inflammation, activation of the immune system, autonomic dysfunction, impaired function of the hypothalamic-pituitary-adrenal axis, and neuroendocrine dysregulation have been discussed as underlying biological causes (2). Furthermore, fatigue often occurs after virus infections, the most prominent example being the Epstein-Barr virus (3); more recently it was described that subjects develop fatigue after a COVID-19 infection (4). In exploring the causes of chronic fatigue, some studies showed an association with cytokine changes in the blood; increases in proinflammatory cytokines such as IL-1ß have been associated with fatigue in patients suffering from several chronic diseases (5). A review of the literature on biomarkers of fatigue revealed that most studies found elevated blood IL-6, TNF-α, and CRP concentrations (2). Recent studies suggested dysregulation of the immune system (6), including T-cell immunity (7). However, data are still insufficient and results are not consistent (8).

In view of the common occurrence of fatigue after infection with SARS-CoV-2, it is a phenomenon that is highly relevant both for those affected themselves and from a health economic perspective. However, effective treatment approaches do not yet exist (9), and only supportive treatment of the symptoms adapted to the individual patient is possible. One reason for this is the fact that the biological mechanisms that cause fatigue are largely unknown.

In the present study, we focused on individuals with a mild course of prior COVID-19 disease and evaluated the specific humoral and T-cell responses to SARS-CoV-2 in men and women with and without post-COVID-19 fatigue. Furthermore, we investigated, whether C-reactive protein (CRP) and IL-6 serum levels were associated with fatigue in both sexes.

## Methods

The present study was a single-center study. Patient recruitment was carried out by the local health authorities of the city and district of Augsburg, Southern Germany. All registered residents with a positive SARS-CoV-2 smear up to November 2020 (n=1600) were invited in writing to participate in the voluntary study.

At the time of the examination, the positive smear had to be at least 14 days ago and the quarantine had to be lifted. In the period from 11/2020 to 05/2021, a total of 525 eligible persons were examined at the University Hospital Augsburg. Of these, 463 study participants were treated exclusively on an outpatient basis. In addition to a physical examination with a focus on vascular complications, demographic data, pre-existing conditions, pre-medication and risk factors were collected using a standardized questionnaire. Furthermore, health-related quality of life and the mental condition of the study participants were evaluated using validated questionnaires. Finally, blood was taken from each study participant and analyzed.

A positive vote of the ethics committee of the Ludwig-Maximilians-Universität München was available (No. 20-735) and written informed consent was obtained from each study participant.

For this analysis, only unvaccinated study participants whose acute COVID-19 disease occurred more than 90 days ago were included (n=283; 131 men and 152 women). We excluded participants with missing values on fatigue-score (n=2). Furthermore, for the analysis regarding the immunologic markers, we also excluded participants with a history of depression (8 men, 14 women). Finally, there were missing values in the biomarker measurements, so the analyses were partly based on different numbers; reference is made to this at the appropriate place.

### Data Collection

In a personal interview and a self-administered questionnaire, information on medical history, smoking habits, medication use, and socioeconomic status was collected by study nurses. Study participants also underwent a medical examination and blood sampling (non-fasting). Schooling was classified into two categories, low (<=10 years of schooling) and high (>10 years of schooling). Marital status was categorized as married (yes/no). When recording previous diseases and risk factors, “I don’t know” statements were added to “no” statements. Prior cardiovascular disease was defined as a history of myocardial infarction or stroke.

### Fatigue Assessment

We used the Fatigue Assessment Scale (FAS), a reliable and valid tool for assessing fatigue symptoms (10). The FAS consists of 10 items with answer options from “never” (1) to “always” (5), whereby fatigue levels between 10 (minimum score) and 50 (maximum score) are calculated. Persons with scores below 22 are indicated to have no fatigue, subjects with scores between 22 and 35 can be classified as having moderate fatigue, and subjects with scores above 35 have a high level of fatigue (11). In the present analysis, persons with scores above 21 were classified as having fatigue.

### Assessment of Immunologic Characteristics

Levels of anti-SARS-CoV2-spike IgG antibodies were measured by an enzyme-linked immunosorbent assay (ELISA) using the Elecsys immunoassay (Roche Diagnostics, Mannheim, Germany) following the manufacturer’s specifications. A positive result was indicated by a value >0.4 U/ml and the upper measurement limit was set to 2,500 U/ml. Due to the manufacturer the upper limit of the test is 250 U/mL. For SARS-CoV2S antibodies > 250 U/ml, a dilution of 1:10 was automatically performed. In case of dilution by the instrument, the software automatically takes the dilution into account when calculating the sample concentration. For the exploration of the SARS-CoV2-specific T-cell response, ex vivo ELISpot/FLUOROspot assays were conducted using the interferon-γ (IFN-γ) and interleukin-2 (IL-2) SARS-CoV-iSpot kit from Autoimmun Diagnostika (AID GmbH, Straßberg, Germany). Peripheral blood mononuclear cells (PBMC) drawn in citrate tubes were isolated on the day of sample collection or the following day by Ficoll-Paque (GE Healthcare, Munich, Germany) and seeded at a density of 2 × 105 cells per well in AIM-V medium in duplicate. Cells were stimulated for 18 hours with the AID-SARS-CoV-2 peptide library containing peptides from the S, N, M, and O proteins and the CD28 antibody, or cells were left unstimulated with the CD28 antibody alone as a negative control. To confirm specific responses to SARS-CoV-2 peptides, cells were stimulated in parallel with peptide libraries cross-reactive with all coronaviruses (PAN) and with libraries specific for cytomegalovirus, Epstein-Barr virus, and influenza virus (CEF). Pokeweed mitogen was used as a positive control. We counted the number of spots by using the iSpot Reader from AID GmbH. Samples were excluded if the positive control Pokeweed Mitogen had fewer than 50 spot-forming units/2 × 105 cells or if the unstimulated control values were >10 or >20 spot-forming units/2 × 105 cells for IFNγ or IL-2, respectively. Positive responses were defined by stimulation index (stimulated spot-forming units/2 × 105 cells divided by unstimulated spot-forming units/2 × 105 cells) as clearly positive with a stimulation index of ≥7 and clearly negative with a stimulation index of ≤3. Samples with values that fell in between were defined as negative unless the unstimulated control value was >2 staining units/2 × 105 cells and the stimulation index was >3, as described by the manufacturer (12). In the present manuscript, measurements are shown as stimulation index (SI) values and not as spot-forming units due to the different background levels in the IFN-γ and IL-2 assays.

### Blood Parameters

C-reactive protein (CRP; reference range 0-0.5 mg/dL) was measured by an article-enhanced immunological turbidity test, where the aggregates are determined turbidimetrically (Cobas instrument, Roche Diagnostics, Mannheim, Germany). Interleukin-6 (IL-6; reference range <15 pg/mL) was measured from serum samples using the Immunological ECLIA test (ElektroChemiLuminescence ImmunoAssay) on Cobas instrument (Roche Diagnostics, Mannheim, Germany). Serum creatinine was analyzed by the Jaffe method. Glucose levels were measured in serum using the GLUC3 assay on a Cobas c702 instrument (Roche Diagnostics GmbH, Mannheim, Germany).

### Statistical Analysis

Quantitative data are reported as median and interquartile range (IQR) and groups were compared by Wilcoxon Rank Sum Test. Categorical data are presented as numbers (%) and differences between groups were tested by Chi-square or Fisher’s exact test. A p-value < 0.05 was considered statistically significant. Statistical analysis was conducted using SAS version 9.4 and R version 4.1.0.

## Results

Median time between acute illness with COVID-19 and examination day was 279 (IQR 179; 325) days. The participants were on average 47.0 years (SD 15.3 years) old, and 54.1% were female. Women more significantly suffered from fatigue in comparison to men (47.4% versus 25.6%, p=0.0002). A high level of fatigue (fatigue score above 35) was present in 5 (3.9%) men and 15 (9.9%) women, a moderate level of fatigue (fatigue score between 22 and 35) in 28 (21.7%) men and 57 (37.5%) women; 96 (74.4%) men and 80 (52.6%) women had no fatigue (fatigue score below 22).

The sex-specific baseline characteristics and clinical features dichotomized in participants with and without fatigue are shown in **Table 1**. In both males and females, almost no differences could be seen regarding age, BMI, disease history, and routine blood parameters.

**Table 1:** Sex-specific characteristics of study participants (frequencies and, in parentheses, percentages or median values and, interquartile range in parentheses), by the presence of post-COVID-19 chronic fatigue syndrome (yes/no).

	Fatigue no	Fatigue yes	p-value*
**WOMEN**	n = 80	n = 72	
Age (years)	46 (32; 57.5)	47 (34;55.5)	0.9573
Body mass index (kg/m²)	23.0 (21.2; 26.2)	24.2 (21.3; 27.7)	0.2399
Hypertension	15 (18.8)	9 (12.5)	0.3742
Diabetes mellitus	4 (5.0)	0	–
Depression	4 (5.0)	10 (13.9)	0.0899
Cardiovascular disease	5 (6.25)	3 (4.2)	0.7223
School education	55 (68.8)	44 (61.1)	0.3945
Marital status	47 (58.8)	49 (68.1)	0.2445
Current smoker	24 (30.0)	27 (37.5)	0.3905
Time since acute infection (days)	272.0 (186.5;315.5)	269.5 (151.5;337.0)	0.9971
White blood cell count* (/nl)	6.62 (5.74;7.61)	6.38 (5.40;7.29)	0.2829
Hematocrit^a^ (l/L)	39.3 (37.8;40.6)	39.9 (38.0;41.5)	0.1305
Hemoglobin^a^ (g/L)	134 (126;138.5)	135 (130;141)	0.2120
Platelets^a^ (/nl)	243 (213;287)	240 (211;274)	0.6160
Glucose ^b^ (mg/dL)	86 (76.5;103)	86 (80;99)	0.6464
Creatinine^c^ (mg/dL)	0.76 (0.68;0.82)	0.74 (0.67;0.81)	0.4038
CRP^d^ (mg/dL)	0.09 (0.06; 0.17)	0.09 (0.06; 0.22)	0.8213
IL-6^e^ (pg/mL)	2.50 (2.50; 3.50)	3.50 (2.50; 3.50)	0.0031
**MEN**	n = 96	n = 33	
Age (years)	52.5 (36.5; 60)	51 (37; 57)	0.8332
Body mass index (kg/m²)	26.6 (24.6; 29.4)	26.60 (23.5; 29.0)	0.5690
Hypertension	23 (24.0)	7 (21.2)	0.8157
Diabetes mellitus	4 (4.2)	2 (6.1)	0.6454
Depression	5 (5.2)	3 (9.1)	0.4208
Cardiovascular Disease	5 (5.2)	4 (12.1)	0.2325
School education	72 (75.0)	26 (78.8)	0.8143
Marital status	67 (69.8)	25 (75.8)	0.6564
Current smoker	43 (44.8)	16 (48.5)	0.8398
Time since acute infection (days)	289.5 (185.5;336.5)	287.0 (185.0;316.0)	0.4197
White blood cell count* (/nl)	6.17 (5.48;7.52)	6.61 (5.46;7.27)	0.9496
Hematocrit^a^ (l/L)	43.5 (41.4;44.9)	44.0 (41.7;45.5)	0.5693
Hemoglobin^a^ (g/L)	150.5 (143;157)	152 (143;157)	0.9868
Platelets^a^ (/nl)	223.5 (198;261)	229 (174;248)	0.7771
Glucose^b^ (mg/dL)	89.0 (81;99)	90.5 (83.5;105.0)	0.4031
Creatinine^c^ (mg/dL)	0.94 (0.87;1.04)	0.99 (0.84;1.06)	0.8028
CRP^d^ (mg/dL)	0.08 (0.06; 0.16)	0.09 (0.06;0.20)	0.2984
IL-6^e^ (pg/mL)	2.80 (2.50;3.50)	3.09 (2.50;3.50)	0.5790

a Based on 94 men without fatigue, and 71 women with fatigue;

b based on 32 men with fatigue, 95 men without fatigue and 71 women with fatigue;

c based on 93 men without fatigue, and 79 women without and 71 with fatigue;

d based on 95 men without fatigue, and 79 women without and 71 women with fatigue;

e based on 77 men without and 26 men with fatigue, and 67 women without and 63 women with fatigue; *Wilcoxon Rank Sum Test

### Cellular Immunity

Evaluation of the cellular immune response quantified by a SARS-CoV2 IFN-γ ELISpot assay revealed that IFN-γ producing cells equal or below the cut-off of SI=3 (clearly negative result) were detected in 63 (24.1%) of the 261 individuals tested. SARS-CoV2 IL-2 producing cells equal or below the cut-off of SI=3 were detected in 91 of the 221 (41.2%) subjects with measured values. Altogether in 221 participants both IL-2 and IFN-γ producing cells could be measured; IFN-γ- and IL-2 producing cells equal or under the cut-off of SI=3 were found in 102 (46.2%) participants. Women had more often clearly negative results than men.

**Figure 1:**
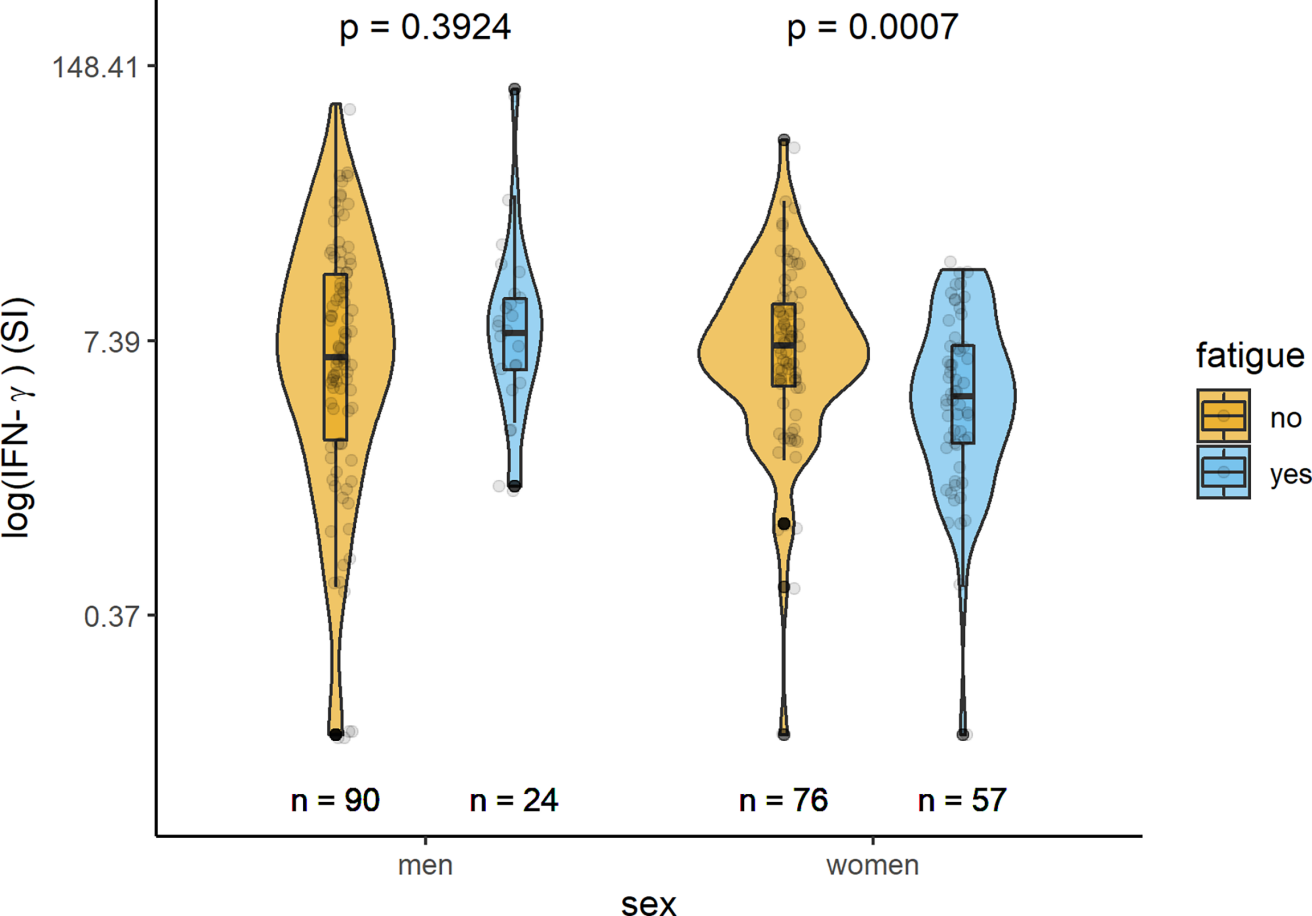
Sex-specific comparison of cells producing IFN-γ measured by ELISpot after stimulation with SARS-CoV-2 antigens (SI values) between persons with and without fatigue on the logarithmic scale.

**Figure 2:**
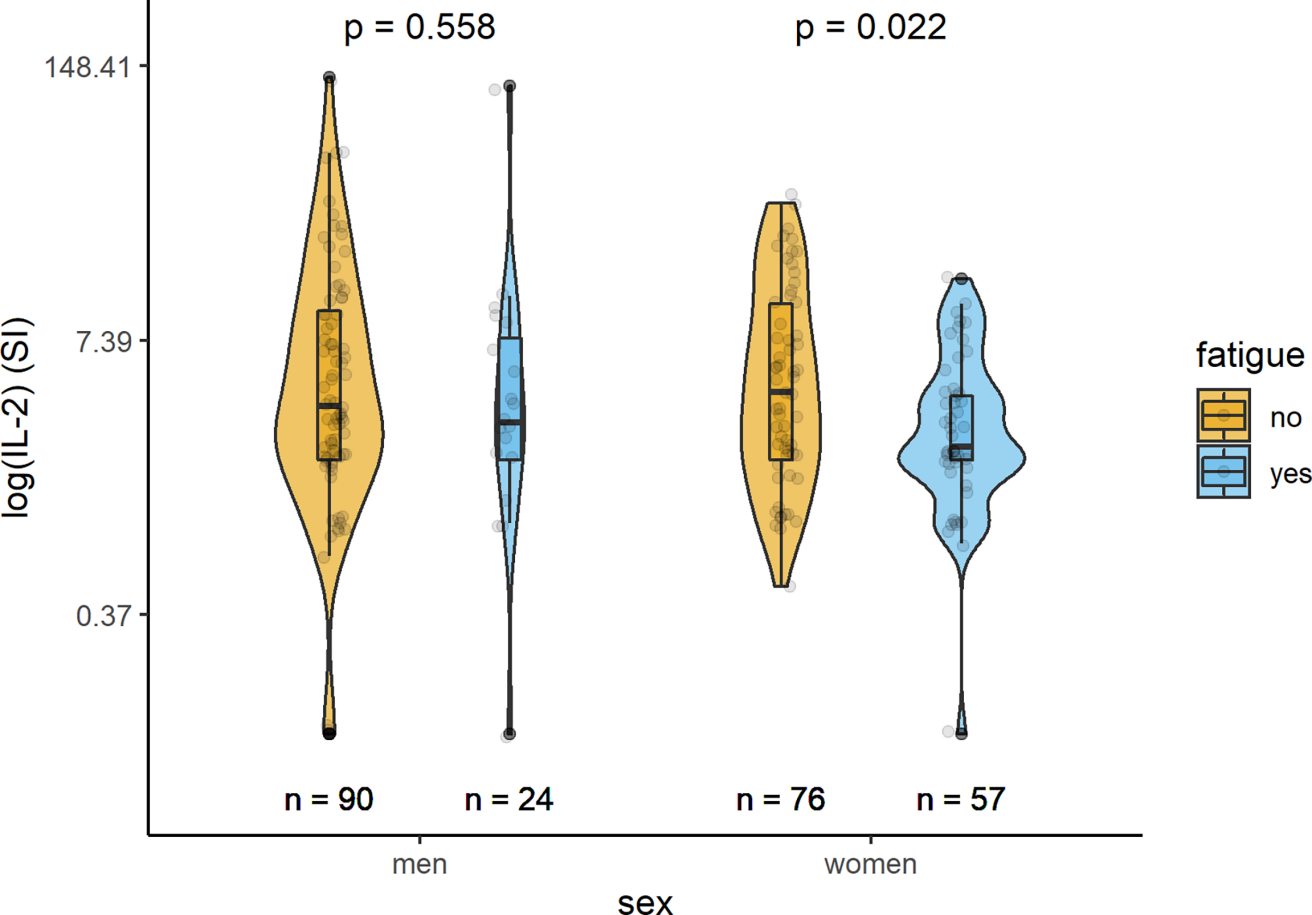
Sex-specific comparison of cells producing IL-2 measured by ELISpot after stimulation with SARS-CoV-2 antigens (SI values) between persons with and without fatigue on the logarithmic scale.

**Figure 3:**
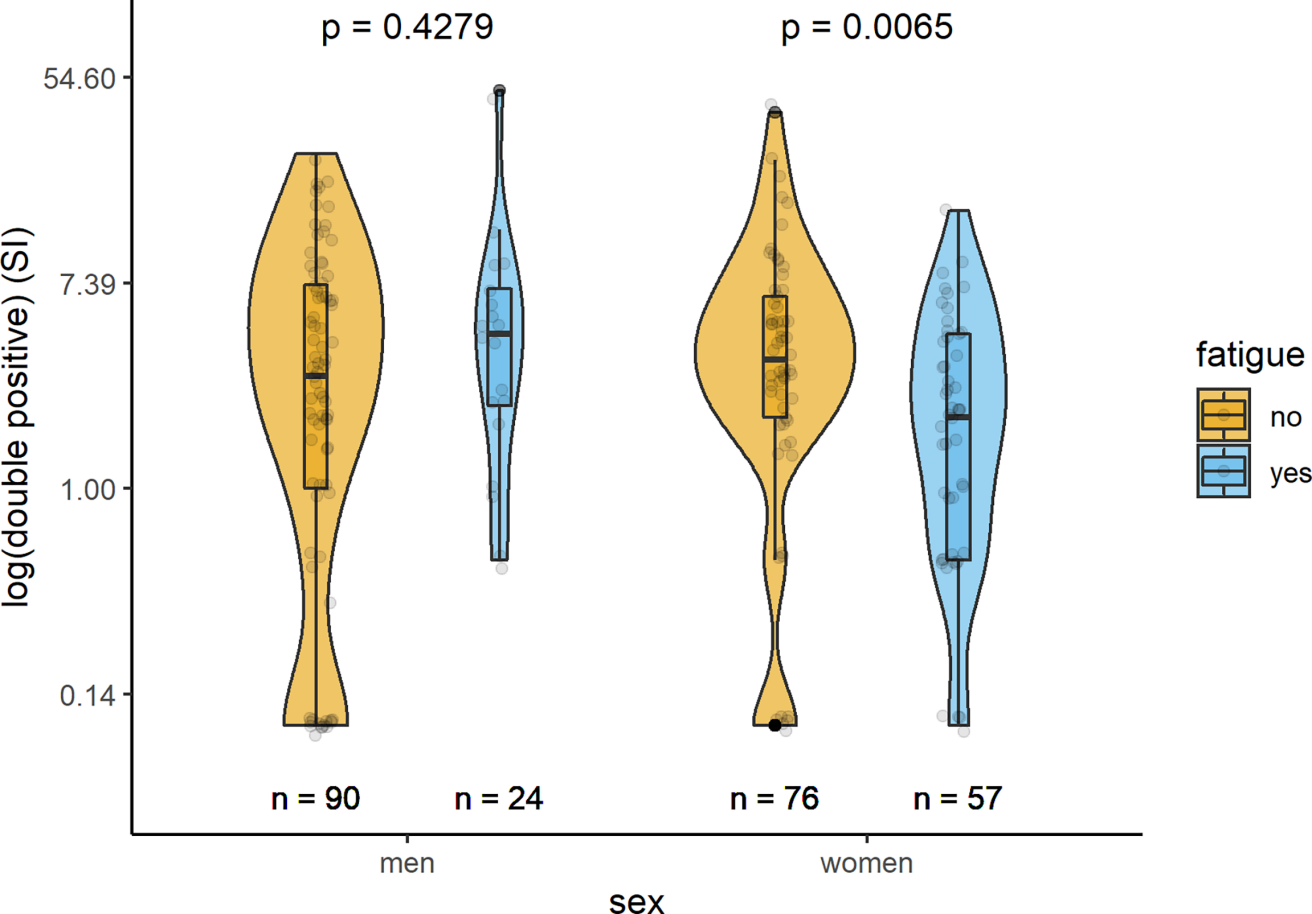
Sex-specific comparison of cells producing both IFN-γ and IL-2 measured by ELISpot after stimulation with SARS-CoV-2 antigens (SI values) between persons with and without fatigue on the logarithmic scale.

In **Table 2** and **Figures 1**–**3**, the results (given as SI) of the comparison of T-cell response between participants with and without fatigue are shown. There were no differences in the immunologic SARS-CoV-2-specific IFN-γ T cell response (IFN-γ, IL-2, or both) between these groups in men. However, measures of T-cell responses significantly differed between women with and without fatigue. Females with fatigue showed a significantly lower number of IFN-γ, IL-2, or both IL-2 and IFNγ producing T-cells in comparison with females without fatigue.

**Table 2:** Number of IFN-γ, IL-2, and both IFN-γ and IL-2 producing cells, measured by ELISpot after stimulation of PBMC *in vitro* with a SARS-CoV-2-specific peptide library (SI values) in men and women with and without post-COVID-19 fatigue.

		Study participants		
	total	w/o fatique	with fatique	
MEN	N=114	n=90	n=24	p-value*
IFN-γ	114	6.15 (2.5; 15.25)	8 (5.375; 11.625)	0.392
IL-2	98	3.6 (2; 10.2)	3 (2; 7.65)	0.558
IFN-γ and IL-2	98	3 (1; 7.25)	4.5 (2.25; 7)	0.428
WOMEN	N=133	n=76	n=57	p-value
IFN-γ	133	7 (4.5; 11)	4 (2.4; 7)	0.001
IL-2	110	4.2 (2; 11)	2.3 (2; 4)	0.022
IFN-γ and IL-2	110	3.5 (2; 6.5)	2 (0.5; 4.5)	0.007

Cells were stimulated for 18 h with the AID SARS-CoV-2 peptide library—containing peptides from the S, N, M, and O proteins—and CD28 antibody, or left unstimulated with CD28 antibody alone as a negative control. Stimulation was performed without cytokines as described by AID.

*Wilcoxon Rank Sum Test.

Significant p-values are given in bold letters.

### Humoral Immunity

**Figure 4:**
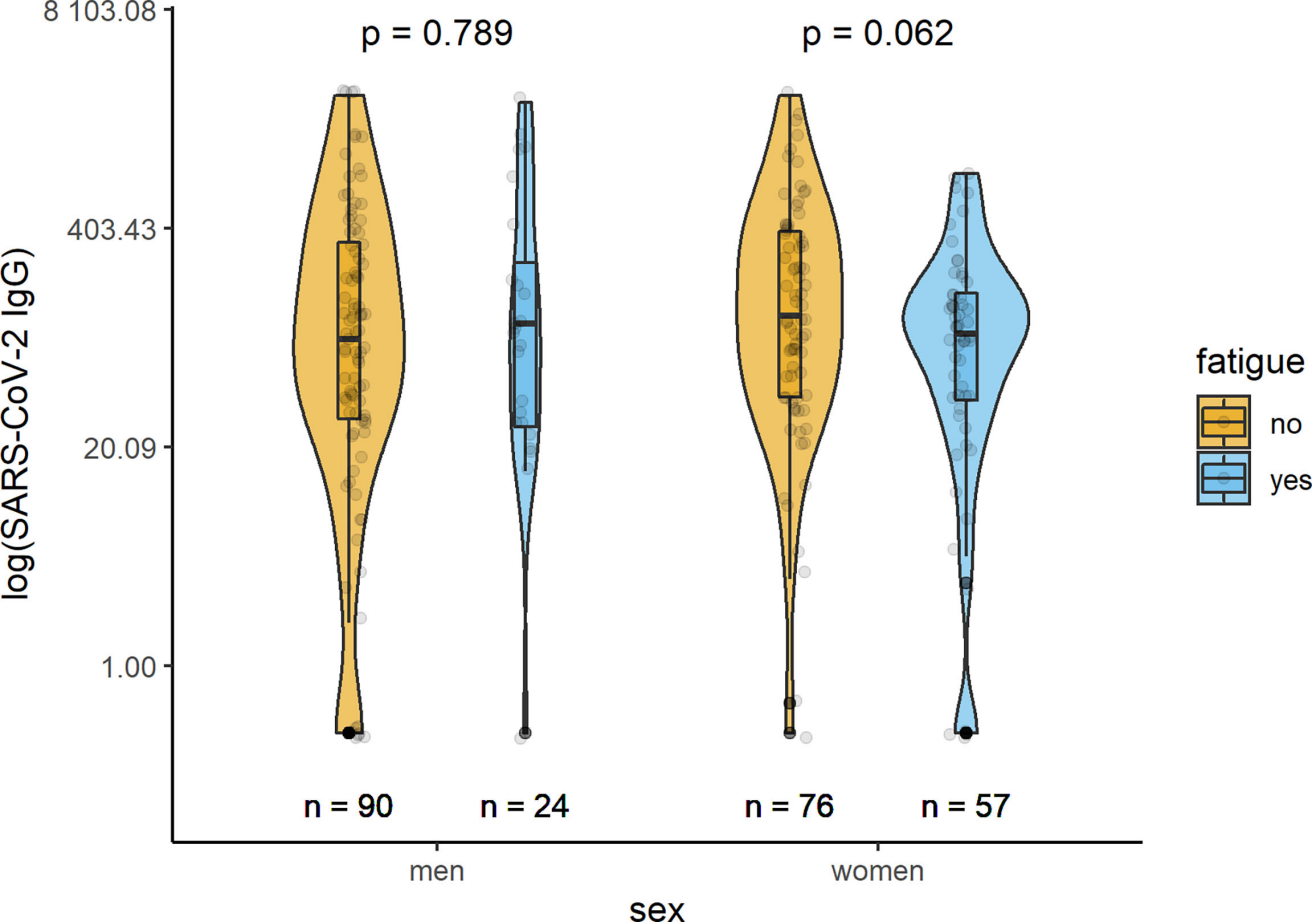
Sex-specific comparison of SARS-CoV-2 IgG antibodies (U/ml) between persons with and without fatigue on the logarithmic scale.

In all but one examined participants, specific IgG antibodies in serum samples were measured at a median time of 279 (IQR 181; 326) days after acute infection with SARS-CoV-2. Median levels of IgG antibodies were 107.5 (34.9;311.5) U/ml in the total sample, 89.5 (27.0;306.0) U/ml in men, and 116.0 (39.7; 322.0) U/ml in women. In both sexes, serum levels of anti-SARS-CoV2-spike IgG antibodies did not differ significantly between participants with or without fatigue (**Figure 4**). The IgG antibody levels in men with and without fatigue were 109 (26.45;268.25) U/mL and 88.1 (29.65;332.75) U/mL (p=0.789), respectively. In women the corresponding levels were 95.1 (38.2;166) U/mL and 121.5 (39.78;385) U/mL (p-value 0.062), respectively.

### Inflammatory Markers

Median serum CRP concentrations were similar for males and females with and without fatigue [men: 0.09 (0.06; 0.20) mg/dL versus 0.08 (0.06; 0.16) mg/dL, p=0.2984; women 0.09 (0.06; 0.22) mg/dL versus 0.09 (0.06; 0.17) mg/dL, p=0.8213]. However, while there were no significant differences in median serum IL-6 levels in men with and without fatigue [with fatigue: 3.09 (2.50;3.50) pg/mL, without fatigue: 2.80 (2.50;3.50) pg/mL; p-value 0.579], the levels differed significantly in women [with fatigue: 3.50 (2.50; 3.50) pg/mL, without fatigue: 2.50 (2.50; 3.50) pg/mL; p-value 0.0031] (**Table 1**).

## Discussion

The present study could show that recovered men and women treated as outpatients during a COVID-19 disease maintained both types of immune response several months after the acute infection. Fatigue was more common in women. When comparing individuals with post-COVID fatigue with individuals without it, T-lymphocyte response between both groups did not differ in men, while in women significantly lower IFN-γ and IL-2 responses could be shown. The humoral SARS-CoV2 IgG antibodies did not significantly differ between the two groups, neither in men nor in women. Thus, it seems possible that alterations in the sex-specific responses to the virus could be related to the development of fatigue after infection.

Levels of IgG antibodies specifically directed against the spike protein of SARS-CoV-2 were detectable in all participants in our study. This is in accordance with data from another study, that reported a percentage of 90% spike IgG-positive convalescents half a year after COVID-19 (13). In addition, persistent seropositivity after 7 and 4 months in patients with mild or moderate COVID-19 have also been reported by other studies (14, 15). In the present study, IgG antibodies did not significantly differ between individuals with and without fatigue, neither in men nor in women. So far, no similar study regarding post-COVID-19 fatigue has been conducted, making comparisons to other investigations difficult. One former study on long-term symptoms after COVID-19 found a weak correlation between long-term loss of taste/smell and low IgA levels at early time points (16), but not with IgG levels during follow-up.

It is well known that women are affected by fatigue much more often (about 3 times as often) than men (17). But, it is still unclear whether infections cause symptoms or those reflect a suppressed immune system (18, 19). Possibly, the effectiveness of an immune response of a person may be involved in the development of fatigue months after the acute infection (3, 20). Thus, we examined the T-cell response against SARS-CoV-2 in men and women with and without fatigue using the ELISpot assay, which predominantly measures immediate responses from effector and effector memory T-cells.

Our findings confirm and extend previous study results showing that SARS-CoV-2-specific IL-2 and/or IFN-γ-producing cells were present in blood samples from recovered individuals who had milder COVID-19 courses (13, 21, 22). Although the ELISpot assay may not be suitable to measure the longevity of T-cell responses, we could show that specific T-cell responses appeared to be maintained for a median time of 9 (IQR 6;11) months after the onset of disease. When comparing women with and without fatigue, we found lower T-cell responses measured by ELISpot in affected women; this was not the case in men. This applied to the IFN-γ (acute) and IL-2 (memory) reaction of pathogen-specifically activated T cells against SARS-CoV-2. Detection of IFN-γ-producing T cells is a sign of immunity to intracellular pathogens and previous studies have shown that SARS-CoV-2-specific IFN-γ-producing T cells to be of the phenotype CD4+ (T helper 1 [Th1]) or cytotoxic phenotype CD8+ (23, 24). It could be hypothesized that a decreased specific T cell response in women could be involved in the development of post-COVID-19 fatigue. However, the immune responses to a viral disease are much more extensive and complex, and depend on a number of factors (25, 26). The medium-term T-lymphocyte responses and antibody levels in recovered patients investigated in our study in connection with the manifestation of fatigue represent only one part of the immune response to SARS-CoV-2. Further studies are needed to investigate the role of cellular and humoral immunity in this context.

Low-grade systemic inflammation also seems to play a role in the pathophysiology of chronic fatigue syndrome, but prior findings were not consistent (2, 27). We found a significantly higher IL-6 level in women with fatigue in comparison to the no-fatigue group, while in men there were no differences. However, the median IL-6 levels observed in our study were very low and within the reference range that is found in healthy individuals. Therefore, it is difficult to draw any conclusions in terms of biological significance from this finding. 

Although female sex is in particular associated with the occurrence of the fatigue syndrome, so far no study carried out sex-specific analyses on this issue in connection with post-COVID-19 fatigue. Further studies are needed to explore possible sex-specific mechanisms involved in individuals with fatigue and to identify possible factors mediating sex differences in immune responses.

The present study has several limitations. First, we included mainly middle-aged men and women of German nationality, thus, generalizability to other age groups and ethnicities is limited. Second, only persons with a mild COVID-19 infection were included and thus, the results are not transferrable to severe cases of infection. Third, only T-cell responses *via* ELISpot were measured in this study and no information on SARS-CoV-2-specific central memory CD4+ or CD8+ T-cells were available. Fourth, only CRP and IL-6 were available as inflammatory markers. Furthermore, no high-sensitive CRP values were measured in this study. Fifth, biomarkers and fatigue were measured only once after a mild COVID-19 infection and no information on them at the timepoint of the acute disease was available. In addition, we do not have information on smear test results and measurements at the time of acute infection. Sixth, prior studies could show that IL-6 serum levels could be affected by hormonal/menstrual cycle (28). Unfortunately, we were not able to take this into account in our study, as no data on hormonal/menstrual cycles were collected in the study. Seventh, due to the low number of cases with high level of fatigue, especially in men, a further analysis by stratification into moderate and severe fatigue was not possible in our study.In conclusion, the present results showed that a specific immune response against SARS-CoV-2 is present in men and women after a mild or moderate COVID-19 infection. It seems that the extent of T-cell response in women but not men is associated with the occurrence of fatigue. More studies are needed to understand the sex-specific role of cellular immunity regarding the manifestation of fatigue after a COVID-19 disease.

## Data Availability Statement

The datasets generated during and/or analyzed during the current study are not publicly available due to data protection aspects but are available in an anonymized form from the corresponding author on reasonable request.

## Ethics Statement

The studies involving human participants were reviewed and approved by Ethics committee of the Ludwig-Maximilians-Universität München (No. 20-735). The patients/participants provided their written informed consent to participate in this study.

## Author Contributions

CM and IK conceived the study. CM performed the statistical analysis and drafted the manuscript. YG, TW, VL, AH-D, and JL contributed to data aquisition, interpretation of the results, and revised the manuscript. All authors contributed to the article and approved the submitted version.

## Funding

This work was funded from the Bavarian state funding for SARSCoV-2 research projects 2020.

## Conflict of Interest

The authors declare that the research was conducted in the absence of any commercial or financial relationships that could be construed as a potential conflict of interest.

## Publisher’s Note

All claims expressed in this article are solely those of the authors and do not necessarily represent those of their affiliated organizations, or those of the publisher, the editors and the reviewers. Any product that may be evaluated in this article, or claim that may be made by its manufacturer, is not guaranteed or endorsed by the publisher.

## References

[1] Pawlikowska T Chalder T Hirsch SR Wallace P Wright DJM Wessely S. Population-Based Study of Fatigue and Psychological Distress. Br Med J (1994) 308(6931):763–6. doi: 10.1136/bmj.308.6931.763 PMC2539651 7908238

[2] Klimas NG Broderick G Fletcher MA. Biomarkers for Chronic Fatigue. Brain Behav Immun (2012) 26(8):1202–10. doi: 10.1016/j.bbi.2012.06.006 PMC5373648 22732129

[3] Hickie I Davenport T Wakefield D Vollmer-Conna U Cameron B Vernon SD. Post-Infective and Chronic Fatigue Syndromes Precipitated by Viral and non-Viral Pathogens: Prospective Cohort Study. Br Med J (2006) 333(7568):575–8. doi: 10.1136/bmj.38933.585764.AE PMC1569956 16950834

[4] Ganesh R Grach SL Ghosh AK Bierle DM Salonen BR Collins NM. The Female-Predominant Persistent Immune Dysregulation of the Post-COVID Syndrome. Mayo Clin Proc (2022) 97(3):454–64. doi: 10.1016/j.mayocp.2021.11.033 PMC8817110 35135695

[5] Roerink ME van der Schaaf ME Dinarello CA Knoop H van der Meer JWM. Interleukin-1 as a Mediator of Fatigue in Disease: A Narrative Review. J Neuroinflamm (2017) 14:16. doi: 10.1186/s12974-017-0796-7 PMC5251329 28109186

[6] Milivojevic M Che XY Bateman L Cheng A Garcia BA Hornig M. Plasma Proteomic Profiling Suggests an Association Between Antigen Driven Clonal B Cell Expansion and ME/CFS. PloS One (2020) 15(7):e0236148. doi: 10.1371/journal.pone.0236148 32692761 PMC7373296

[7] Karlsson AC Humbert M Buggert M. The Known Unknowns of T Cell Immunity to COVID-19. Sci Immunol (2020) 5(53):eabe8063. doi: 10.1126/sciimmunol.abe8063 33208380

[8] Kutlubaev MA Duncan FH Mead GE. Biological Correlates of Post-Stroke Fatigue: A Systematic Review. Acta Neurol Scandinavica (2012) 125(4):219–27. doi: 10.1111/j.1600-0404.2011.01618.x 22070461

[9] Wu S Kutlubaev MA Chun HY Cowey E Pollock A Macleod MR. Interventions for Post-Stroke Fatigue. Cochrane Database Syst Rev (2015) 2015(7):CD007030. doi: 10.1002/14651858.CD007030.pub3 PMC7387276 26133313

[10] Michielsen HJ De Vries J Van Heck GL. Psychometric Qualities of a Brief Self-Rated Fatigue Measure The Fatigue Assessment Scale. J Psychosomatic Res (2003) 54(4):345–52. doi: 10.1016/S0022-3999(02)00392-6 12670612

[11] Available at: https://www.ildcare.nl/index.php/how-to-use-the-fas-fatigue-assessment-scale/.

[12] Dennehy KM Loll E Dhillon C Classen JM Warm TD Schuierer L. Comparison of the Development of SARS-Coronavirus-2-Specific Cellular Immunity, and Central Memory CD4+ T-Cell Responses Following Infection Versus Vaccination. Vaccines (2021) 9(12):1439. doi: 10.3390/vaccines9121439 34960185 PMC8707815

[13] Dan JM Mateus J Kato Y Hastie KM Yu ED Faliti CE. Immunological Memory to SARS-CoV-2 Assessed for Up to 8 Months After Infection. Science (2021) 371(6529):587–+. doi: 10.1126/science.abf4063 PMC7919858 33408181

[14] Glück V Grobecker S Tydykov L Salzberger B Glück T Weidlich T. SARS-CoV-2-Directed Antibodies Persist for More Than Six Months in a Cohort With Mild to Moderate COVID-19. Infection (2021) 49(4):739–46. doi: 10.1007/s15010-021-01598-6 PMC7944246 33689159

[15] Wheatley AK Juno JA Wang JJ Selva KJ Reynaldi A Tan HX. Evolution of Immune Responses to SARS-CoV-2 in Mild-Moderate COVID-19. Nat Commun (2021) 12(1):1162. doi: 10.1038/s41467-021-21444-5 33608522 PMC7896046

[16] Rank A Tzortzini A Kling E Schmid C Claus R Loll E. One Year After Mild COVID-19: The Majority of Patients Maintain Specific Immunity, But One in Four Still Suffer From Long-Term Symptoms. J Clin Med (2021) 10(15):3305. doi: 10.3390/jcm10153305 34362088 PMC8347559

[17] Bateman L Bested AC Bonilla HF Chheda BV Chu L Curtin JM. Myalgic Encephalomyelitis/Chronic Fatigue Syndrome: Essentials of Diagnosis and Management. Mayo Clin Proc (2021) 96(11):2861–78. doi: 10.1016/j.mayocp.2021.07.004 34454716

[18] Komaroff AL. Inflammation Correlates With Symptoms in Chronic Fatigue Syndrome. Proc Natl Acad Sci USA (2017) 114(34):8914–6. doi: 10.1073/pnas.1712475114 PMC5576849 28811366

[19] Komaroff AL Cho TA. Role of Infection and Neurologic Dysfunction in Chronic Fatigue Syndrome. Semin Neurol (2011) 31(3):325–37. doi: 10.1055/s-0031-1287654 21964849

[20] Rydyznski Moderbacher C Ramirez SI Dan JM Grifoni A Hastie KM Weiskopf D. Antigen-Specific Adaptive Immunity to SARS-CoV-2 in Acute COVID-19 and Associations With Age and Disease Severity. Cell (2020) 183(4):996–1012.e19. doi: 10.1016/j.cell.2020.09.038 33010815 PMC7494270

[21] Zuo JM Dowell AC Pearce H Verma K Long HM Begum J. Robust SARS-CoV-2-Specific T Cell Immunity is Maintained at 6 Months Following Primary Infection (Vol 22, Pg 620, 2021). Nat Immunol (2021) 22(7):928–. doi: 10.1038/s41590-021-00957-7 PMC8134969 34017126

[22] Breton G Mendoza P Hagglof T Oliveira TY Schaefer-Babajew D Gaebler C. Persistent Cellular Immunity to SARS-CoV-2 Infection. J Exp Med (2021) 218(4):e20202515. doi: 10.1084/jem.20202515 33533915 PMC7845919

[23] Grifoni A Weiskopf D Ramirez SI Mateus J Dan JM Moderbacher CR. Targets of T Cell Responses to SARS-CoV-2 Coronavirus in Humans With COVID-19 Disease and Unexposed Individuals. Cell (2020) 181(7):1489–501 e15. doi: 10.1016/j.cell.2020.05.015 32473127 PMC7237901

[24] Sekine T Perez-Potti A Rivera-Ballesteros O Stralin K Gorin JB Olsson A. Robust T Cell Immunity in Convalescent Individuals With Asymptomatic or Mild COVID-19. Cell (2020) 183(1):158–68. e14. doi: 10.1016/j.cell.2020.08.017 32979941 PMC7427556

[25] Seow J Graham C Merrick B Acors S Pickering S Steel KJA. Longitudinal Observation and Decline of Neutralizing Antibody Responses in the Three Months Following SARS-CoV-2 Infection in Humans. Nat Microbiol (2020) 5(12):1598–607. doi: 10.1038/s41564-020-00813-8 PMC7610833 33106674

[26] Robbiani DF Gaebler C Muecksch F Lorenzi JCC Wang Z Cho A. Convergent Antibody Responses to SARS-CoV-2 in Convalescent Individuals. Nature (2020) 584(7821):437–42. doi: 10.1038/s41586-020-2456-9 PMC7442695 32555388

[27] Sulheim D Fagermoen E Winger A Andersen AM Godang K Muller F. Disease Mechanisms and Clonidine Treatment in Adolescent Chronic Fatigue Syndrome: A Combined Cross-Sectional and Randomized Clinical Trial. JAMA Pediatr (2014) 168(4):351–60. doi: 10.1001/jamapediatrics.2013.4647 24493300

[28] Jilma B Dirnberger E Löscher I Rumplmayr A Hildebrandt J Eichler HG. Menstrual Cycle-Associated Changes in Blood Levels of Interleukin-6, Alpha1 Acid Glycoprotein, and C-Reactive Protein. J Lab Clin Med (1997) 130(1):69–75. doi: 10.1016/S0022-2143(97)90060-3 9242368

